# Associations of gallbladder and gallstone parameters with clinical outcomes in patients with cirrhosis

**DOI:** 10.2478/jtim-2022-0076

**Published:** 2023-03-19

**Authors:** Min Ding, Yue Yin, Xueying Wang, Menghua Zhu, Shixue Xu, Le Wang, Fangfang Yi, Cyriac Abby Philips, Fernando Gomes Romeiro, Xingshun Qi

**Affiliations:** Liver Cirrhosis Study Group, Department of Gastroenterology, the General Hospital of Northern Theater Command (formerly General Hospital of Shenyang Military Area), Shenyang 110840, Liaoning Province, China; Postgraduate College, China Medical University, Shenyang 110840, Liaoning Province, China; Postgraduate College, Jinzhou Medical University, Jinzhou 121001, Liaoning Province, China; Clinical and Translational Hepatology & Monarch Liver Laboratory, the Liver Institute Center of Excellence in Gastrointestinal Sciences, Rajagiri Hospital, Kochi 682028, India; Gastroenterology Division, Department of Internal Medicine, Botucatu Medical School, Universidade Estadual Paulista (UNESP), Botucatu 18608917, Brazil

**Keywords:** liver cirrhosis, gallbladder, gallstone, survival, decompensation

## Abstract

**Background:**

Morphologic changes in the gallbladder and gallstones are common in cirrhotic patients, but their associations with outcomes of cirrhotic patients are unclear.

**Methods:**

We retrospectively enrolled 206 cirrhotic patients and measured their gallbladder length and width, gallbladder wall thickness, presence of gallstones, and gallstones’ length and width in axial contrast-enhanced computed tomography (CT) images. X-tile software was utilized to calculate the optimal cutoff values of these parameters for evaluating survival and hepatic decompensation events in the cirrhosis group. Their associations with survival were explored by Cox regression analyses and Kaplan–Meier curve analyses. Their associations with hepatic decompensation events were evaluated by competing risk analyses and Nelson-Aalen cumulative risk curve analyses where death was a competing event.

**Results:**

Cirrhotic patients with gallbladder length < 72 mm had a significantly higher cumulative survival rate than those with a length of ≥ 72 mm (*P* = 0.049 by log-rank test), but gallbladder width, gallbladder wall thickness, presence of gallstones, and gallstones’ length and width were not significantly associated with survival (*P* = 0.10, *P* = 0.14, *P* = 0.97, *P* = 0.73, and *P* = 0.73 by log-rank tests, respectively). Cirrhotic patients with gallbladder wall thickness < 3.4 mm had a significantly lower cumulative rate of hepatic decompensation events than those with a wall thickness of ≥ 3.4 mm (*P* = 0.02 by Gray’s test), but gallbladder length and width, presence of gallstones, and gallstones’ length and width were not significantly associated with hepatic decompensation events (*P* = 0.15, *P* = 0.15, *P* = 0.54, *P* = 0.76, and *P* = 0.54 by Gray’s tests, respectively).

**Conclusion:**

Changes in gallbladder length and gallbladder wall thickness, rather than gallstone parameters, may be in parallel with the long-term outcomes of cirrhotic patients.

## Introduction

Liver cirrhosis, the end stage of chronic liver diseases, carries a high morbidity and mortality.^[1, 2, 3]^ It can lead to many lethal complications, including gastroesophageal variceal bleeding, ascites, hepatic encephalopathy, and jaundice.^[4]^ According to the Global Burden of Disease Study 2017, liver cirrhosis caused more than 1.32 million deaths, which constituted 2.4% of total deaths globally.^[5]^

Gallbladder, a pear-shaped organ, stores and concentrates bile between meals.^[6]^ Hemodynamically, gallbladder venous drainage is through the portal venous system, which subsequently flows into the inferior vena cava.^[7, 8]^ In liver cirrhosis, cholecystic venous outflow tract can be impaired due to increased portal venous pressure, resulting in gallbladder congestion manifesting as changes in gallbladder length and width and gallbladder wall thickness in the axial images.^[9, 10]^ On the other hand, gallbladder motility may be reduced due to portal hypertension in liver cirrhosis.^[11]^ Consequently, liver cirrhosis is associated with a high risk of gallstones, and the prevalence of gallstones is twice higher in patients with liver cirrhosis than in the general population.^[12, 13, 14]^ However, the associations of gallbladder morphologic changes and gallstones with the outcomes of patients with cirrhosis remain unclear. For this reason, the present study mainly aimed to investigate the associations of various gallbladder and gallstone parameters, including gallbladder length and width, gallbladder wall thickness, presence of gallstones, and gallstones’ length and width, with the longterm survival and development of hepatic decompensation events in patients with cirrhosis. Besides, their correlations with the Child-Pugh score and the model for end-stage liver disease (MELD) score were also evaluated.

## Methods

This study was carried out following the rules of the 1975 Declaration of Helsinki and was approved by the Medical Ethical Committee of our hospital (Approval No. Y [2022] 071). Patients’ written informed consents had been waived by the Medical Ethical Committee of our hospital due to the retrospective nature of this study.

### Study design

This retrospective study was performed on the basis of our prospective database in which a total of 570 patients with cirrhosis were consecutively admitted to the Department of Gastroenterology of our hospital and underwent abdominal contrast-enhanced computed tomography (CT) or magnetic resonance imaging (MRI) scans from December 2014 to November 2021. A diagnosis of liver cirrhosis was mainly based on clinical manifestations including gastrointestinal bleeding, ascites, and hepatic encephalopathy, laboratory tests including liver dysfunction, decreased serum albumin, and coagulation dysfunction, imaging features including liver morphology changes with splenomegaly on abdominal CT scans, and liver histological features including pseudolobule, if necessary.

Inclusion criteria were as follows: (1) patients who were diagnosed with liver cirrhosis and (2) patients who performed abdominal contrast-enhanced CT scans during hospitalization. Exclusion criteria were as follows: (1) a history of malignancy; (2) gallbladder and gallstone parameters could not be sufficiently evaluated on CT images; (3) a history of cholecystectomy; and (4) acute or chronic cholecystitis was diagnosed based on disease history, clinical presentations, and/or biochemical signs. Notably, patients with asymptomatic gallstones were not excluded.

Additionally, by reviewing both electronic medical records and imaging reports system, all patients without a history of chronic liver disease and hepatocellular carcinoma or other malignancies who were consecutively admitted to the Department of Gastroenterology of our hospital and underwent abdominal contrast-enhanced CT between January 2020 and October 2021 were selected as the control group.

### Clinical data collection

All patients were subjected to complete clinical evaluation. Clinical data at admission were collected as follows: age, gender, red blood cell (RBC), white blood cell (WBC), platelets count (PLT), total bilirubin (TBIL), albumin (ALB), alanine aminotransferase (ALT), γ-glutamine transferase (GGT), serum creatinine (Scr), serum sodium (Na), prothrombin time (PT), and international normalized ratio (INR). The Child-Pugh score and the MELD score were calculated.

### CT images

**Figure 1:**
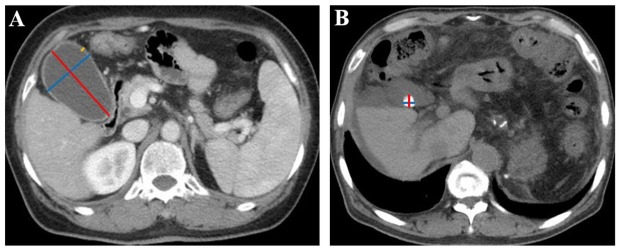
Gallbladder and gallstone parameters measured in CT images. (A) Measurement of gallbladder parameters in a 63-year-old woman with liver cirrhosis. The red line represents the maximum gallbladder length, the blue line represents the maximum gallbladder width, and the yellow line represents the maximum gallbladder wall thickness. The maximum gallbladder length was 77.6 mm, the maximum gallbladder width was 49.0 mm, and the maximum gallbladder wall thickness was 3.5 mm. (B) Measurement of gallstone parameters in a 65-year-old man with liver cirrhosis. The red line represents the maximum gallstone length, and the blue line represents the maximum gallstone width. The maximum gallstone length was 12.7 mm, and the maximum gallstone width was 12.4 mm. CT: computed tomography.

Two investigators independently reviewed all available CT images of each included patient, selected the specific layers, where the maximum length and width of gallbladder, the maximum thickness of the gallbladder wall, and the maximum length and width of gallstones were obtained, and then measured these parameters according to the standard methods as shown in Figure 1. A disagreement between them was resolved by discussing with another investigator to determine the most appropriate layer where the value measured should be maximal as the final value. Notably, all patients are routinely requested to be fasting before undergoing abdominal contrast-enhanced CT or MRI scan.

### Follow-up

All enrolled patients were followed by reviewing inpatient and outpatient medical records and telephone visits. The last follow-up date was December 9, 2021. We recorded the dates of hepatic decompensation events and deaths during follow-up. Hepatic decompensation events evaluated in this study included gastrointestinal bleeding, ascites, hepatic encephalopathy, and jaundice.^[15, 16, 17, 18]^

### Statistical analyses

First, continuous variables were expressed as the mean ± standard deviation or median (range) and compared using nonparametric Mann-Whitney *U* test. Categorical variables were expressed as frequency (percentage) and compared using the *χ*^2^ test. Second, the correlations of various gallbladder and gallstone parameters with the Child-Pugh score and the MELD score were analyzed by Spearman’s rank correlation tests, and the Spearman’s rank correlation coefficients (*rs*) were calculated. Third, X-tile software was utilized to calculate the optimal cutoff values of gallbladder and gallstone parameters for evaluating the long-term overall survival and hepatic decompensation events. Fourth, univariate Cox regression analyses were performed to explore the associations of gallbladder and gallstone parameters with survival and multivariate Cox regression analyses were performed by adjusting for age, sex, and the Child-Pugh score to identify which parameter was an independent predictor of survival. Hazard ratios (HRs) and their 95% confidence intervals (CIs) were calculated. Cumulative survival rates were evaluated by Kaplan-Meier curve analyses and compared by log-rank tests. Fifth, competing risk analyses were performed to analyze the associations of these parameters with hepatic decompensation events during follow-up, where death was considered a competing event. Sub-distribution hazard ratios (sHRs) and their 95% CIs were calculated. Cumulative rates of hepatic decompensation events were evaluated by Nelson-Aalen cumulative risk curve analyses and compared by Gray’s tests. All statistical analyses were performed on IBM Statistical Package for the Social Sciences (SPSS) version 25.0 (IBM Corp, Armonk, NY, USA), X-tile version 3.6.1 (Yale University, New Haven, CT, USA), and R version 4.1.2 (R Foundation for Statistical Computing, Vienna, Austria) with the packages ggplot2, survival, survminer, and cmprsk. A two-tailed *P* value < 0.05 was considered statistically significant.

## Results

### Study population

**Figure 2:**
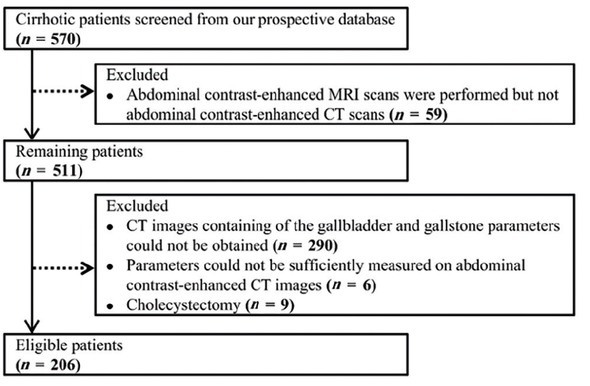
Flowchart of cirrhotic patients’ selection. MRI: magnetic resonance imaging.

Overall, 206 patients with cirrhosis were included (Figure 2). Median age was 56 years (28–89) and 142 (69%) patients were male (Table 1). During a median follow-up duration of 2.25 years (0.09–6.03), 54 patients developed gastrointestinal bleeding, 42 developed ascites, 10 developed hepatic encephalopathy, 29 died, and none underwent liver transplantation. Causes of death were related to liver diseases (*n* = 23), non-liver diseases (*n* = 1), and were unknown (*n* = 5).

**Table 1:** Differences between cirrhosis group and non-cirrhosis group

**Variables**	**No. pts**	**Cirrhosis group**	**No. pts**	**Non-cirrhosis group**	***P* value**
Demographics					
Age, yr	206	56 (28–89)	104	58 (19–81)	0.22
Male, *n* (%)	206	142 (69)	104	67 (64)	0.42
Laboratory tests					
RBC, ×1012/L	206	3.48 (1.24–5.20)	99	4.06 ± 0.68	< 0.001
WBC, ×109/L	206	3.20 (0.70–19.60)	99	6.00 (2.20–15.30)	< 0.001
PLT, ×109/L	206	80 (24–646)	99	233 (25–759)	< 0.001
TBIL, μmol/L	206	19.40 (5.70–216.50)	99	10.10 (3.10–300.70)	< 0.001
ALB, g/L	205	33.95 ± 6.72	99	37.96 ± 4.66	< 0.001
ALT, U/L	206	24.52 (4.23–1465.50)	99	15.42 (2.68–215.29)	< 0.001
GGT, U/L	206	36.35 (8.23–1283.02)	99	22.50 (5.97–651.14)	< 0.001
Scr, μmol/L	203	63.43 (14.80–130.40)	99	68.17 (22.22–436.80)	0.03
Na, mmol/L	206	138.90 (118.00–151.00)	98	140.05 (123.80–147.00)	< 0.001
PT, s	205	15.70 (12.60–28.00)	99	13.52 ± 0.84	< 0.001
INR	205	1.26 (0.99–2.77)	99	1.02 (0.84–1.27)	< 0.001
Gallbladder and gallstone parameters					
Gallbladder length, mm	206	61.07 ± 19.24	104	56.71 ± 18.40	0.06
Gallbladder width, mm	206	32.07 ± 9.21	104	29.62 ± 7.29	0.03
Gallbladder wall thickness, mm	201	3.30 (1.44–7.30)	99	2.50 (1.31–6.28)	< 0.001
Gallstones, *n* (%)	206	39 (19)	104	3 (3)	< 0.001
Gallstones^’^ length, mm	39	6.90 (1.80–22.40)	3	11.17 (1.79–30.34)	< 0.001
Gallbladders^’^ width, mm	39	8.30 (1.70–29.78)	3	11.31 (4.02–13.26)	< 0.001

Continuous data that were normally distributed were expressed as mean ± standard deviation, and those that were skewed were expressed as median (range). pts: numbers of patients; RBC: red blood cell; WBC: white blood cell; PLT: platelets count; TBIL: total bilirubin; ALB: albumin; ALT: alanine aminotransferase; GGT: γ-glutamine transferase; Scr: serum creatinine; Na: serum sodium; PT: prothrombin time; INR: international normalized ratio.

### Cirrhosis versus non-cirrhosis

Overall, 104 patients with non-cirrhosis were selected as the control group (Supplementary Figure 1). Patients with cirrhosis had longer gallbladder and wider gallbladder, thicker gallbladder wall, and higher prevalence of gallstones than those with non-cirrhosis (Table 1).

### Correlations of gallbladder and gallstone parameters with the Child-Pugh score and the MELD score in cirrhosis

Spearman’s rank correlation tests demonstrated that gallbladder width significantly correlated with the MELD score (*P* = 0.03, *rs* = 0.148) and gallbladder wall thickness significantly correlated with the Child-Pugh score (*P* = 0.005, *rs* = 0.196) and the MELD score (*P* = 0.002, *rs* = 0.212). However, gallbladder length, presence of gallstones, gallstones’ length, and gallstones’ width did not significantly correlate with the Child-Pugh score or the MELD score (Table 2).

**Table 2:** Correlations of gallbladder and gallstone parameters with the Child-Pugh score and the MELD score in cirrhosis

**Variables**	**Child-Pugh score**		**MELD score**	
	***r*_s_**	***P* value**	***r*_s_**	***P* value**
Gallbladder length	–0.020	0.77	0.012	0.87
Gallbladder width	0.021	0.76	0.148	0.03
Gallbladder wall thickness	0.196	< 0.01	0.212	< 0.01
Gallstones	0.018	0.80	0.080	0.26
Gallstones^’^ length	0.025	0.88	0.066	0.35
Gallstones^’^ width	0.104	0.53	0.077	0.27

MELD: model for end-stage liver disease.

### Associations of gallbladder and gallstone parameters with survival in cirrhosis

The optimal cutoff values of gallbladder length, gallbladder width, gallbladder wall thickness, gallstones’ length, and gallstones’ width for predicting survival were 72.0, 38.6, 3.6, 1.9, and 2.0 mm, respectively.

Univariate Cox regression analyses demonstrated that gallbladder length < 72.0 mm, gallbladder width < 38.6 mm, gallbladder wall thickness < 3.6 mm, absence of gallstones, gallstones’ length < 1.9 mm, and gallstones’ width < 2.0 mm were not significant predictors of survival in cirrhosis. However, after adjusting for age, sex, and the Child-Pugh score, multivariate Cox regression analyses demonstrated that gallbladder width < 38.6 mm was an independent predictor of survival (HR = 3.01, 95% CI: 1.28–7.06; *P* = 0.01), but not gallbladder length < 72.0 mm, gallbladder wall thickness < 3.6 mm, absence of gallstones, gallstones’ length < 1.9 mm, or gallstones’ width < 2.0 mm (Table 3).

**Table 3:** Cox regression analyses regarding the associations of gallbladder and gallstone parameters with survival in cirrhosis

**Variables**	**Univariate analyses**			**Multivariate analyses^a^**		
	**HR**	**95% CI**	***P* value**	**HR**	**95% CI**	***P* value**
Gallbladder length < 72.0 *versus* ≥ 72.0 mm	2.07	0.99–4.34	0.05	1.99	0.94–4.22	0.07
Gallbladder width < 38.6 *versus* ≥ 38.6 mm	1.93	0.88–4.25	0.10	3.01	1.28–7.06	0.01
Gallbladder wall thickness < 3.6 *versus* ≥ 3.6 mm	1.72	0.83–3.56	0.15	1.09	0.49–2.42	0.84
Without gallstones *versus* with gallstones	0.98	0.40–2.41	0.97	0.63	0.24–1.63	0.34
Gallstones^’^ length < 1.9 *versus* ≥ 1.9 mm	0.85	0.32–2.22	0.74	0.51	0.18–1.43	0.20
Gallstones^’^ width < 2.0 *versus* ≥ 2.0 mm	0.85	0.32–2.22	0.74	0.51	0.18–1.43	0.20

^a^Multivariate analyses by adjusting for age, sex, and the Child-Pugh score. HR: hazard ratio; CI: confidence interval.

**Figure 3:**
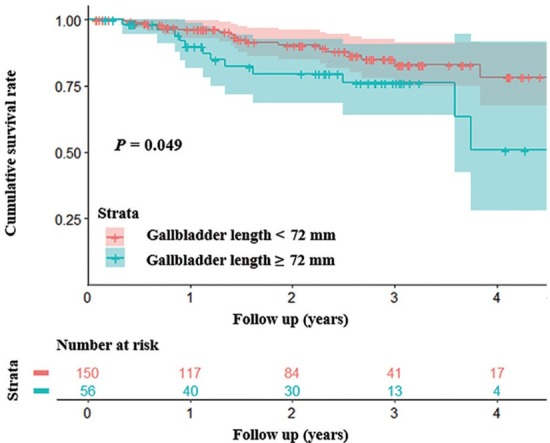
Kaplan-Meier curve analysis demonstrating that patients with gallbladder length < 72 mm had a significantly higher cumulative survival rate than those with a length of ≥ 72 mm (*P* = 0.049 by log-rank test).

Kaplan-Meier curve analysis demonstrated that patients with gallbladder length < 72.0 mm had a significantly higher cumulative survival rate than those with a length of ≥ 72.0 mm (*P* = 0.049 by log-rank test) (Figure 3). But there was no significant difference in the cumulative survival rate between patients with gallbladder width < 38.6 mm *versus* those with a width of ≥ 38.6 mm (*P* = 0.10 by log-rank test), those with gallbladder wall thickness < 3.6 mm *versus* patients with a wall thickness of ≥ 3.6 mm (*P* = 0.14 by log-rank test), those without gallstones *versus* those with gallstones (*P* = 0.97 by log-rank test), those with gallstones’ length < 1.9 mm *versus* those with a length of ≥ 1.9 mm (*P* = 0.73 by log-rank test), or those with gallstones’ width < 2.0 mm *versus* those with a width of ≥ 2.0 mm (*P* = 0.73 by log-rank test).

### Associations of gallbladder and gallstone parameters with hepatic decompensation events in cirrhosis

The optimal cutoff values of gallbladder length, gallbladder width, gallbladder wall thickness, gallstones’ length, and gallstones’ width for predicting hepatic decompensation events were 55.8, 31.1, 3.4, 1.9, and 1.7 mm, respectively. Competing risk analyses demonstrated that gallbladder wall thickness < 3.4 mm was significantly associated with decreased risk of hepatic decompensation events (sHR = 1.62, 95% CI: 1.09–2.41; *P* = 0.02), but not gallbladder length < 55.8 mm, gallbladder width < 31.1 mm, absence of gallstones, gallstones’ length < 1.9 mm, or gallstones’ width < 1.7 mm (Table 4).

**Table 4:** Competing risk analyses regarding the associations of gallbladder and gallstone parameters with hepatic decompensation events in cirrhosis

**Variables**	**sHR**	**95% CI**	***P* value**
Gallbladder length < 55.8 *versus* ≥ 55.8 mm	0.75	0.50–1.11	0.15
Gallbladder width < 31.1 *versus* ≥ 31.1 mm	1.35	0.91–2.01	0.14
Gallbladder wall thickness < 3.4 *versus* ≥ 3.4 mm	1.62	1.09–2.41	0.02
Without gallstones *versus* with gallstones	1.16	0.75–1.81	0.51
Gallstones^’^ length < 1.9 *versus* ≥ 1.9 mm	1.08	0.68–1.72	0.74
Gallstones^’^ width < 1.7 *versus* ≥ 1.7 mm	1.16	0.75–1.81	0.51

sHR: sub-distribution hazard ratio; CI: confidence interval.

**Figure 4:**
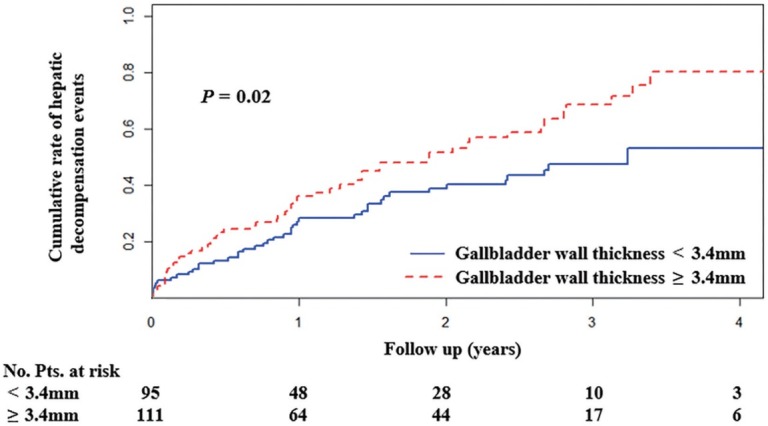
Nelson-Aalen cumulative risk curve analysis demonstrating that patients with gallbladder wall thickness < 3.4 mm had a significantly lower cumulative rate of hepatic decompensation events than those with a thickness of ≥ 3.4 mm (*P* = 0.02 by Gray’s test). Pts: patients.

Nelson–Aalen cumulative risk curve analysis demonstrated that patients with gallbladder wall thickness < 3.4 mm had a significantly lower cumulative rate of hepatic decompensation events than those with a wall thickness of ≥ 3.4 mm (*P* = 0.02 by Gray’s test) (Figure 4). But there was no significant difference in the cumulative rate of hepatic decompensation events between patients with gallbladder length < 55.8 mm *versus* those with a length of ≥ 55.8 mm (*P* = 0.15 by Gray’s test), those with gallbladder width < 31.1 mm *versus* those with a width of ≥ 31.1 mm (*P* = 0.15 by Gray’s test), those without gallstones *versus* those with gallstones (*P* = 0.54 by Gray’s test), those with gallstones’ length < 1.9 mm *versus* those with a length of ≥ 1.9 mm (*P* = 0.76 by Gray’s test), or those with gallstones’ width < 1.7 mm *versus* those with a width of ≥ 1.7 mm (*P* = 0.54 by Gray’s test).

## Discussion

Our study found that patients with cirrhosis had longer and wider gallbladder and thicker gallbladder wall than those without, after excluding the possibilities of gallbladder morphologic changes caused by acute or chronic cholecystitis. Abnormal gallbladder morphology is related to hypoproteinemia caused by reduced liver synthetic function in cirrhotic patients.^[19]^ Hypoproteinemia can lead to a decrease of colloid osmotic pressure, and then the formation of ascites, in which the gallbladder remains immersed for a long time, enlarging the gallbladder size and thickening the gallbladder wall.^[20]^ More importantly, this might be primarily due to gallbladder congestion caused by portal hypertension in liver cirrhosis. Indeed, as the portal pressure increases, multiple hepatic decompensation events will develop. For example, esophageal varices develop and progress, thereby causing variceal rupture and massive gastrointestinal bleeding,^[21,22]^ and the vascular hydrostatic pressure in the abdominal viscera increases, thereby decreasing tissue fluid reabsorption and then aggravating the occurrence and grade of ascites.^[23]^ Recently, the association of gallbladder wall thickness with hepatic decompensation has been explored in some cross-sectional studies.^[7,24, 25, 26]^ Elkerdawy *et al*.^[7]^ found that gallbladder wall thickness was associated with the presence of esophageal varices in patients with cirrhosis. Mohammadi *et al*.^[25]^ demonstrated that thickened gallbladder wall was highly predictive for the presence of ascites in liver cirrhosis. Our cross-sectional data supported that gallbladder wall thickness significantly correlated with the Child-Pugh and MELD scores, which are well-known prognostic factors in patients with cirrhosis,^[27,28]^ and that gallbladder width also significantly correlated with the MELD score. Notably, all previous studies reported only cross-sectional data without long-term outcomes, and therefore, they could not provide any evidence regarding the associations of gallbladder wall thickness with the development and progression of decompensation events and death during follow-up. Moreover, they employed ultrasound to measure gallbladder changes. By comparison, our study employed abdominal contrast-enhanced CT scans, which could provide more accurate and reproducible data, followed patients with cirrhosis for a median duration of 2.25 years, and further demonstrated that gallbladder wall thickness and gallbladder length were positively associated with the risk of hepatic decompensation events and death, respectively. Additionally, the normal gallbladder length ranges 80–120 mm and the upper limit of normal of gallbladder wall thickness is 3 mm.^[29]^ We also identified that the optimal cutoff values of gallbladder length and width for predicting the risk of death should be ≥ 72.0 and ≥ 38.6 mm, respectively. The optimal cutoff value of gallbladder wall thickness for predicting the risk of hepatic decompensation events should be ≥ 3.4 mm. The changes in gallbladder morphology in cirrhosis may reflect the severity of liver cirrhosis itself to a certain extent and can provide observable indicators for the outcomes of cirrhotic patients.

Our study also found a significantly higher prevalence of gallstones in patients with cirrhosis than in those without. This might be because cirrhotic patients with portal hypertension often have reduced gallbladder motility,^[30,31]^ patients with advanced liver cirrhosis may present with autonomic neuropathy leading to sphincter of Oddi dysfunction and gallbladder emptying impairment,^[32,33]^ and those with hypersplenism develop chronic hemolysis resulting in the formation of black pigment gallstones.^[34]^ Previous studies have shown that gallstone disease was associated with high risk of all-cause death and disease-specific death, including cardiovascular disease and cancer-related mortality.^[35]^ However, Ruhl and Everhart^[36]^ reported that gallstone disease was not related to the mortality from digestive diseases, in which chronic liver disease accounted for nearly half of all deaths from digestive diseases. Despite a higher prevalence of gallstones in patients with cirrhosis than in those without,^[37, 38, 39]^ our study further showed no significant association between the presence and size of gallstones and the outcomes of patients with cirrhosis. This may be due to a small proportion of gallbladder stones in our patients. Notably, we should acknowledge that some gallstones could not be clearly observed on abdominal CT images, and transabdominal ultrasonography should be the first-line approach for diagnosing gallstones.^[40]^

Our study had several features. First, the study population was from our prospectively established database of liver cirrhosis and was regularly followed. Second, the data measured by CT were more objective and reproducible. Third, various gallbladder and gallstone parameters measured by abdominal contrast-enhanced CT scans were carefully collected to identify the differences between patients with and without cirrhosis, evaluate their correlations with the severity of liver dysfunction in patients with cirrhosis, and predict hepatic decompensation events and long-term survival. Our study also had several limitations. First, this retrospective study had a potential bias in patient selection. Second, not all our patients had well-preserved contrast-enhanced CT images to measure gallbladder and gallstone parameters. Third, only the gallbladder length and width obtained in the axial images were employed in our study. By comparison, the gallbladder volume should be an optimal indicator of gallbladder morphology. Fourth, the cutoff values of gallbladder and gallstone parameters calculated in our study might not be readily extrapolated to other patients. Fifth, the rate of agreement in the measurement of gallbladder and gallstone parameters among investigators had not been designed.

In summary, patients with liver cirrhosis often develop changes in gallbladder morphology and gallstones. Importantly, thickened gallbladder wall and increased gallbladder length, but not gallstones, predict their worse outcomes. Further studies should elucidate the impact of dynamic changes of gallbladder-related parameters on the prognosis of patients with cirrhosis.

## Author Contributions

Conceptualization: Xingshun Qi; data collection and revision: Min Ding, Yue Yin, Xueying Wang, and Xingshun Qi; data analysis and revision: Min Ding, Yue Yin, Menghua Zhu, Fangfang Yi, and Xingshun Qi; methodology and writing: Min Ding, Yue Yin, and Xingshun Qi; critical comments and revision: Min Ding, Yue Yin, Xueying Wang, Menghua Zhu, Shixue Xu, Le Wang, Fangfang Yi, Cyriac Abby Philips, Fernando Gomes Romeiro, and Xingshun Qi; and supervision: Xingshun Qi.

## Supplementary Material

Supplementary Material Details
