# Supplementary figures and images for: Associations of gallbladder and gallstone parameters with clinical outcomes in patients with cirrhosis
Source: J Transl Int Med. 2023 Mar 19;12(3):308–16. doi: 10.2478/jtim-2022-0076 (PMC11285020; doi:10.2478/jtim-2022-0076)

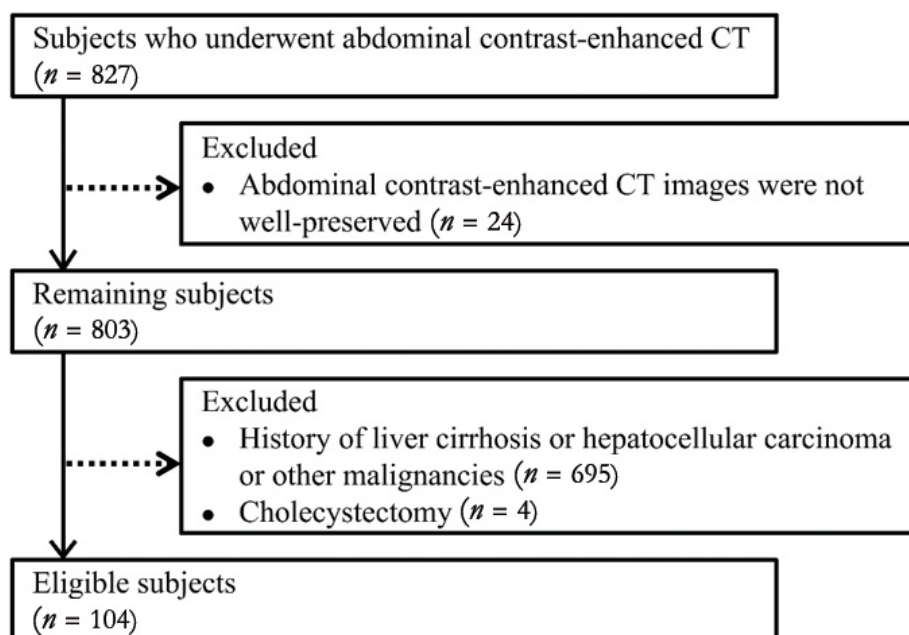

Supplementary Figure 1: Flowchart of control subjects' selection. CT: computed tomography.

Supplement: Supplementary file 1 — Supplementary Material Details [file jtim-2022-0076_sm.pdf]
